# Editorial: Emerging talents in alloimmunity and transplantation: 2022

**DOI:** 10.3389/fimmu.2024.1393026

**Published:** 2024-03-15

**Authors:** Guido Moll, Wai H. Lim, Olaf Penack

**Affiliations:** ^1^ BIH Center for Regenerative Therapies (BCRT) and Berlin-Brandenburg School for Regenerative Therapies (BSRT), Charité Universitätsmedizin Berlin, corporate member of Freie Universität Berlin, Humboldt-Universität zu Berlin, and Berlin Institute of Health (BIH), Berlin, Germany; ^2^ Department of Nephrology and Internal Intensive Care Medicine, Charité Universitätsmedizin Berlin, Berlin, Germany; ^3^ Department of Renal Medicine, Sir Charles Gairdner Hospital, Perth, WA, Australia; ^4^ School of Medical and Health Sciences, Edith Cowan University, Perth, WA, Australia; ^5^ Medical School, University of Western Australia, Perth, WA, Australia; ^6^ Department of Hematology, Oncology and Tumorimmunology, Charité Universitätsmedizin Berlin, Berlin, Germany; ^7^ BIH Biomedical Innovation Academy, Charité Universitätsmedizin Berlin, Berlin, Germany

**Keywords:** alloimmunity, transplantation, rejection, inflammation, cell therapy, immunosuppression, immunomodulation, human leukocyte antigen (HLA)

## Introduction

**Figure 1:**
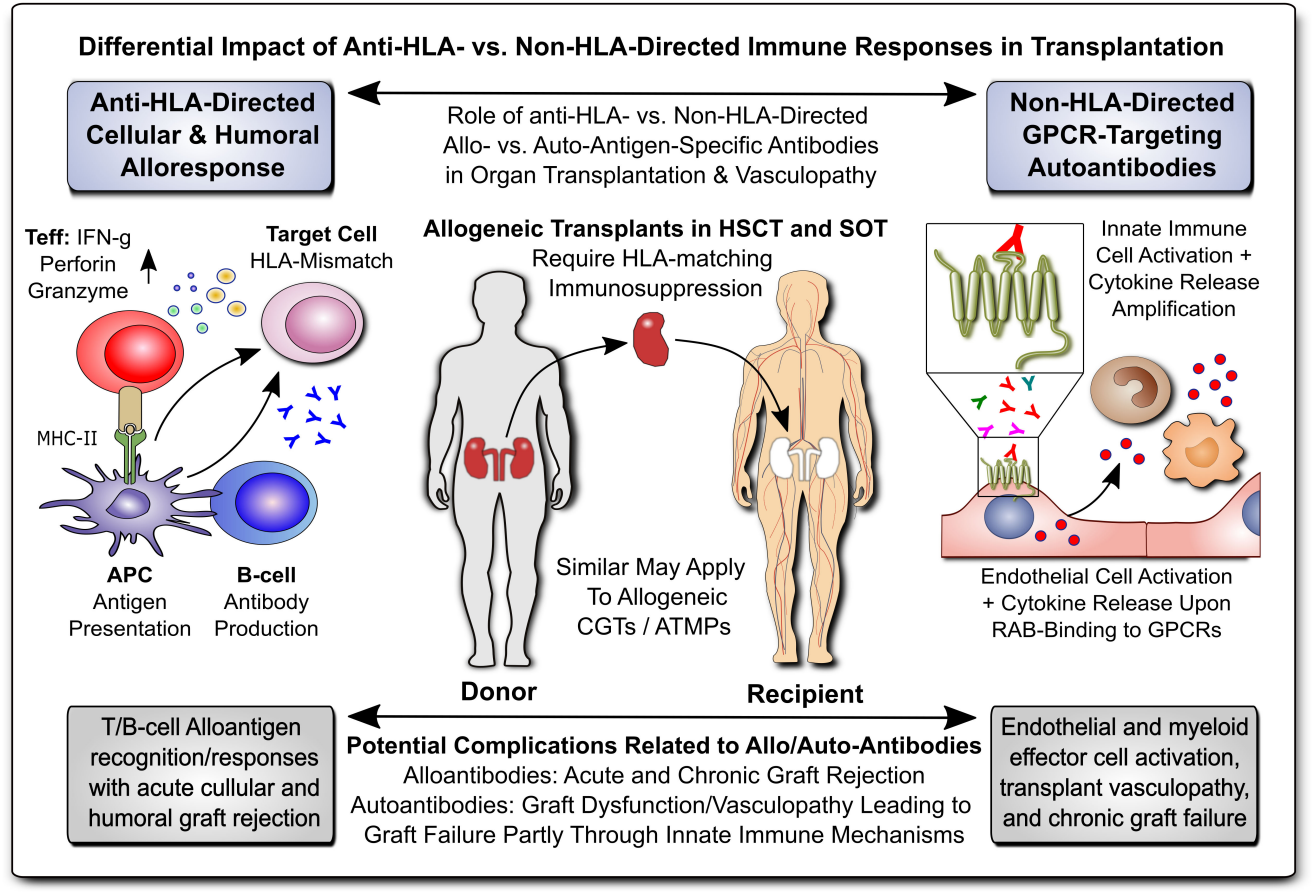
Differential Impact of Anti-HLA- & Non-HLA-Directed Allo- & Auto-Immune Responses in Transplantation. Allogeneic transplants in HSCT and SOT typically require HLA-matching and immunosuppression to prevent allograft rejection through anti-HLA-directed alloantigen-specific immune responses (e.g. T and B cell and alloantibody mediated), with a minor but significant contribution from non-HLA-directed auto-antigen-specific autoantibodies (e.g. GPCR-directed regulatory autoantibodies, RABs). APC, antigen-presenting cell; ATMP, advanced therapy medicinal product; CGT, cell and gene therapy; MHC, major histocompatibility complex; HLA, human leukocyte antigen; HSCT, hematopoietic stem cell transplantation; SOT, solid organ transplantation; Teff and Treg, effector and regulatory T cells; GPCR, G-protein coupled receptor; RAB, regulatory autoantibodies of non-HLA type that are e.g. GPCR-directed, as distinguished from anti-HLA-directed alloantibodies and donor-specific alloantibodies (DSA).

In modern transplant and biomedicine, a proper understanding of both allo- and auto-immune processes is of key importance to minimize acute and chronic graft failure and consecutive rejection/pathology through both cellular and humoral effector mechanisms (**Figure 1**), e.g. cellular and humoral allo-sensitization, alloantigen-reactive T and B cells with the latter producing donor-specific anti-human leukocyte antigen (HLA) alloantibodies (DSA), and also the contribution of autoantibodies (1–7). This field includes many diverse disciplines, but similar underlying principles, such as the need for HLA-matching of donor organs/stem cells with the recipient, prevention and treatment of graft-versus-host disease (GvHD) (7), and the common need for effective immunosuppression (e.g. steroids or tacrolimus, TAC/FK506) (8), as standard immunosuppressive agent for life-long therapy.

Transplantation of donor-tissues and -organs and vascularized composite allografts is commonly summarized under the term solid organ transplantation (SOT), including e.g. hand, kidney, liver, heart, lung, and intestinal transplantation (8, 9). Of similar importance is hematopoietic stem cell transplantation (HSCT), to reconstitute the stem and progenitor cell compartment in the bone marrow, with both SOT and HSCT entailing GvHD as a potential complication (7, 10). In addition, there are numerous novel approaches of cell and gene therapies (CGTs), including advanced therapy medicinal products (ATMPs), in the US and Europe, respectively (10–14). Adjunct technology, such as machine perfusion of donor organs (e.g. hypo- vs. normothermic) and kidney/renal replacement therapies (RRTs) comprise another promising new field (15–18).

## Prior viral infection can promote allograft rejection

Khorki et al. from Cincinnati Children’s Hospital Medical Center in Ohio, USA, contributed the article “Prior viral infection primes cross-reactive CD8+ T cells that respond to mouse heart allograft”. The authors studied the connection between transplant rejection and the presence of high levels of pre-existing memory T cells, in particular virus-specific memory T cells that can drive allograft rejection in allo-MHC animal models and clinical transplantation (1–3, 19–26). They established a mouse model that can track virus-specific, allo-specific, and cross-reactive T cells, revealing that prior infection induces substantial numbers of virus-specific T cells that cross-react to alloantigen, manifesting as early acute rejection of the heart allograft.

## Cross-tissue inflammation in vascularized composite allotransplantation

Shah et al. in collaboration between groups from Yoram Vodovotz (Univ of Pittsburgh, USA) and Vijay Gorantla (Wake Forest Institute of Regenerative Medicine, USA) contributed the article “Peripheral nerve repair is associated with augmented cross-tissue inflammation following vascularized composite allotransplantation (VCA).”. Indeed, VCA with concomitant nerve repair/coadaption (NR) and adjunct TAC immunosuppressive therapy is used to repair traumatic injuries but is often complicated by innate and adaptive immune activation/inflammation spanning multiple tissues (27). The effect of NR on the inflammatory cascade is currently unknown (28–30), although TAC has been reported to enhance NR (31–33). The authors here found that, while NR is considered necessary for graft function, it may result in dysregulated and mis-compartmentalized inflammation post VCA and thus suitable mitigation strategies are needed, pointing at their spatiotemporal bioinformatics pipeline.

## Hypothermic machine perfusion alleviates IRI in intestinal transplantation in pigs

Hou et al. from the Research Institute of Transplant Medicine and Tianjin Key Laboratory for Organ Transplantation at the Tianjin First Central Hospital in China contributed the article: “Hypothermic machine perfusion alleviates ischemia-reperfusion injury (IRI) of intestinal transplantation (IT) in pigs.”. IT is vulnerable to IRI, and due to the limitations of static cold storage (SCS), hypothermic machine perfusion (HMP) is rapidly increasing in popularity (34–40). Here, the authors established a stable intestinal HMP system and demonstrated that HMP could significantly alleviate intestinal IRI and improve the outcome after IT in pigs.

## Microbiota transplantation for irritable bowel syndrome: review and meta-analysis

Wang et al. from the Department of General Surgery in Lanzhou China contributed the article “Fecal microbiota transplantation (FMT) for irritable bowel syndrome (IBS): a systematic review and meta-analysis of randomized controlled trials (RCTs).”. The authors here assessed the safety and efficacy of FMT for patients with IBS in 19 RCTs within their PROSPERO study (CRD42022328377; https://www.crd.york.ac.uk/prospero/). They found that a single stool FMT was effective and safe for patients with IBS. At 3-36 months post initiation of treatment, FMT could significantly reduce the IBS-SSS score and improve the clinical response rate. However, the authors did not find a positive effect of capsule FMT on patients with IBS.

## HDAC6-Inhibition in KTx to modulate adaptative/innate immunity: review article

Zhang et al. from the Institute of Organ Transplantation at Tongji Hospital/Medical College, and the Key Laboratory of Organ Transplantation, Wuhan, China, contributed the article “HDAC6 inhibition: A significant potential regulator and therapeutic option to translate into clinical practice in renal transplantation.”. The enzyme histone deacetylase 6 (HDAC6) plays an essential role in many biological processes and exerts deacetylation-dependent and independent effects on a variety of molecular targets, including modulation of both innate and adaptive immune pathways (41). The authors here reason that HDAC6 inhibitors may be promising therapeutic candidates in kidney transplantation, e.g. to counteract ischemia reperfusion injury (IRI), to induce immune tolerance, to protect against oxidative stress, and to attenuate chronic interstitial fibrosis of the transplanted kidneys (42–45).

## Immunomodulatory allogeneic and autologous cell therapy for COVID-19: review and meta-analysis

Couto et al. in a collaboration between Charité Berlin in Germany and The University of Sao Paulo in Brazil contributed the article “Systematic review and meta-analysis of cell therapy for COVID-19: global clinical trial landscape, published safety/efficacy outcomes, cell product manufacturing and clinical delivery.”. This article provides the largest meta-analysis of 195 registered clinical trials to date. Demographic analysis found that the highest number of trials was conducted in the US, China, and Iran, with the highest number per capita in Israel, Spain, Iran, Australia, and Sweden. The leading cell type was multipotent mesenchymal stromal/stem cells (MSCs) (10, 12–14), which were remarkably heterogenous in their manufacturing and clinical delivery. A pooled analysis of 24 published clinical trials on MSC infusions in COVID-19 found a relative risk reduction for all-cause COVID-19 mortality of RR=0.63 (95% CI 0.46 to 0.85), in alignment with earlier smaller summaries that suggested some clinical benefit (13, 46–52).

## Author contributions

GM: Conceptualization, Data curation, Formal analysis, Funding acquisition, Investigation, Methodology, Project administration, Resources, Software, Supervision, Validation, Visualization, Writing – original draft, Writing – review & editing. WL: Conceptualization, Data curation, Formal analysis, Funding acquisition, Investigation, Methodology, Project administration, Resources, Software, Supervision, Validation, Visualization, Writing – original draft, Writing – review & editing. OP: Conceptualization, Data curation, Formal analysis, Funding acquisition, Investigation, Methodology, Project administration, Resources, Software, Supervision, Validation, Visualization, Writing – original draft, Writing – review & editing.

## References

[1] Rocha PN Plumb TJ Crowley SD Coffman TM. Effector mechanisms in transplant rejection. Immunol Rev. (2003) 196:51–64. doi: 10.1046/j.1600-065X.2003.00090.x 14617197

[2] Ely LK Burrows SR Purcell AW Rossjohn J McCluskey J. T-cells behaving badly: structural insights into alloreactivity and autoimmunity. Curr Opin Immunol. (2008) 20:575–80. doi: 10.1016/j.coi.2008.07.006 18678247

[3] Felix NJ Allen PM. Specificity of T-cell alloreactivity. Nat Rev Immunol. (2007) 7:942–53. doi: 10.1038/nri2200 18007679

[4] Lim WH Ho J Kosmoliaptsis V Sapir-Pichhadze R. Editorial: Future challenges and directions in determining allo-immunity in kidney transplantation. Front Immunol. (2022) 13. doi: 10.3389/fimmu.2022.1013711 PMC9473680 36119031

[5] Cabral-Marques O Moll G Catar R Preuß B Bankamp L Pecher AC. Autoantibodies targeting G protein-coupled receptors: An evolving history in autoimmunity. Report of the 4th international symposium. Autoimmun Rev. (2023) 22:103310. doi: 10.1016/j.autrev.2023.103310 36906052

[6] Moll G Luecht C Gyamfi MA da Fonseca DLM Wang P Zhao H. Autoantibodies from patients with kidney allograft vasculopathy stimulate a proinflammatory switch in endothelial cells and monocytes mediated via GPCR-directed PAR1-TNF-α signaling. Front Immunol. (2023) 14:1289744. doi: 10.3389/fimmu.2023.1289744 37965310 PMC10642342

[7] Penack O Marchetti M Aljurf M Arat M Bonifazi F Duarte RF. Prophylaxis and management of graft-versus-host disease after stem-cell transplantation for haematological Malignancies: updated consensus recommendations of the European Society for Blood and Marrow Transplantation. Lancet Haematology. (2024) 11:e147–59. doi: 10.1016/S2352-3026(23)00342-3 38184001

[8] Roemhild A Otto N M Moll G Abou-El-Enein M Kaiser D Bold G. Regulatory T cells for minimising immune suppression in kidney transplantation: phase I/IIa clinical trial. Bmj. (2020) 371:m3734. doi: 10.1136/bmj.m3734 33087345 PMC7576328

[9] Moll G Dai Z Camara NOS. Editorial: advances in heart transplantation. Front Immunol. (2022) 13:960800. doi: 10.3389/fimmu.2022.960800 35812419 PMC9263967

[10] Ringdén O Moll G Gustafsson B Sadeghi B. Mesenchymal stromal cells for enhancing hematopoietic engraftment and treatment of graft-versus-host disease, hemorrhages and acute respiratory distress syndrome. Front Immunol. (2022) 13:839844. doi: 10.3389/fimmu.2022.839844 35371003 PMC8973075

[11] Goldsobel G von Herrath C Schlickeiser S Brindle N Stähler F Reinke P. RESTORE survey on the public perception of advanced therapies and ATMPs in europe-why the european union should invest more! Front Med (Lausanne). (2021) 8:739987. doi: 10.3389/fmed.2021.739987 34765617 PMC8576137

[12] Moll G Ankrum JA Kamhieh-Milz J Bieback K Ringdén O Volk H-D. Intravascular mesenchymal stromal/stem cell therapy product diversification: time for new clinical guidelines. Trends Mol Med. (2019) 25:149–63. doi: 10.1016/j.molmed.2018.12.006 30711482

[13] Moll G Drzeniek N Kamhieh-Milz J Geissler S Volk H-D Reinke P. MSC therapies for COVID-19: importance of patient coagulopathy, thromboprophylaxis, cell product quality and mode of delivery for treatment safety and efficacy. Front Immunol. (2020) 11:1091. doi: 10.3389/fimmu.2020.01091 32574263 PMC7249852

[14] Moll G Ankrum JA Olson SD Nolta JA. Improved MSC minimal criteria to maximize patient safety: A call to embrace tissue factor and hemocompatibility assessment of MSC products. Stem Cells Trans Med. (2022) 11:2–13. doi: 10.1093/stcltm/szab005 PMC8895495 35641163

[15] Tatum R O’Malley TJ Bodzin AS Tchantchaleishvili V. Machine perfusion of donor organs for transplantation. Artif organs. (2021) 45:682–95. doi: 10.1111/aor.13894 33349946

[16] Basile C Davenport A Mitra S Pal A Stamatialis D Chrysochou C. Frontiers in hemodialysis: Innovations and technological advances. Artif organs. (2021) 45:175–82. doi: 10.1111/aor.13798 32780472

[17] Catar R Moll G Kamhieh-Milz J Luecht C Chen L Zhao H. Expanded hemodialysis therapy ameliorates uremia-induced systemic microinflammation and endothelial dysfunction by modulating VEGF, TNF-α and AP-1 signaling. Front Immunol. (2021) 12:774052. doi: 10.3389/fimmu.2021.774052 34858433 PMC8632537

[18] Zhao H Wu D Gyamfi MA Wang P Luecht C Pfefferkorn AM. Expanded Hemodialysis ameliorates uremia-induced impairment of vasculoprotective KLF2 and concomitant proinflammatory priming of endothelial cells through an ERK/AP1/cFOS-dependent mechanism. Front Immunol. (2023) 14:1209464. doi: 10.3389/fimmu.2023.1209464 37795100 PMC10546407

[19] Burrows SR Silins SL Khanna R Burrows JM Rischmueller M McCluskey J. Cross-reactive memory T cells for Epstein-Barr virus augment the alloresponse to common human leukocyte antigens: degenerate recognition of major histocompatibility complex-bound peptide by T cells and its role in alloreactivity. Eur J Immunol. (1997) 27:1726–36. doi: 10.1002/eji.1830270720 9247584

[20] Heeger PS Greenspan NS Kuhlenschmidt S Dejelo C Hricik DE Schulak JA. Pretransplant frequency of donor-specific, IFN-gamma-producing lymphocytes is a manifestation of immunologic memory and correlates with the risk of posttransplant rejection episodes. J Immunol. (1999) 163:2267–75. doi: 10.4049/jimmunol.163.4.2267 10438971

[21] Augustine JJ Siu DS Clemente MJ Schulak JA Heeger PS Hricik DE. Pre-transplant IFN-gamma ELISPOTs are associated with post-transplant renal function in African American renal transplant recipients. Am J Transplant. (2005) 5:1971–5. doi: 10.1111/j.1600-6143.2005.00958.x 15996247

[22] Archbold JK Macdonald WA Burrows SR Rossjohn J McCluskey J. T-cell allorecognition: a case of mistaken identity or déjà vu? Trends Immunol. (2008) 29:220–6. doi: 10.1016/j.it.2008.02.005 18378495

[23] Macdonald WA Chen Z Gras S Archbold JK Tynan FE Clements CS. T cell allorecognition via molecular mimicry. Immunity. (2009) 31:897–908. doi: 10.1016/j.immuni.2009.09.025 20064448

[24] Zeng G Huang Y Huang Y Lyu Z Lesniak D Randhawa P. Antigen-specificity of T cell infiltrates in biopsies with T cell-mediated rejection and BK polyomavirus viremia: analysis by next generation sequencing. Am J Transplant. (2016) 16:3131–8. doi: 10.1111/ajt.13911 PMC5083170 27273900

[25] Wang Y Singh NK Spear TT Hellman LM Piepenbrink KH McMahan RH. How an alloreactive T-cell receptor achieves peptide and MHC specificity. Proc Natl Acad Sci U.S.A. (2017) 114:E4792–e4801. doi: 10.1073/pnas.1700459114 28572406 PMC5474831

[26] Stranavova L Pelak O Svaton M Hruba P Fronkova E Slavcev A. Heterologous cytomegalovirus and allo-reactivity by shared T cell receptor repertoire in kidney transplantation. Front Immunol. (2019) 10:2549. doi: 10.3389/fimmu.2019.02549 31736968 PMC6834532

[27] Schneeberger S Gorantla VS Van Riet RP Lanzetta M Vereecken P Van Holder C. Atypical acute rejection after hand transplantation. Am J Transplant. (2008) 8:688–96. doi: 10.1111/j.1600-6143.2007.02105.x 18261182

[28] Bueno E Benjamin M-J Sisk G Sampson CE Carty M Pribaz JJ. Rehabilitation following hand transplantation. Handb (N Y). (2014) 9:9–15. doi: 10.1007/s11552-013-9568-8 PMC3928383 24570631

[29] Glaus SW Johnson PJ Mackinnon SE. Clinical strategies to enhance nerve regeneration in composite tissue allotransplantation. Handb Clin. (2011) 27:495–509. doi: 10.1016/j.hcl.2011.07.002 PMC3212838 22051390

[30] Chan KM Gordon T Zochodne DW Power HA. Improving peripheral nerve regeneration: from molecular mechanisms to potential therapeutic targets. Exp Neurol. (2014) 261:826–35. doi: 10.1016/j.expneurol.2014.09.006 25220611

[31] Doolabh VB Mackinnon SE. FK506 accelerates functional recovery following nerve grafting in a rat model. Plast reconstructive Surg. (1999) 103:1928–36. doi: 10.1097/00006534-199906000-00018 10359255

[32] Feng FY Ogden MA Myckatyn TM Grand AG Jensen JN Hunter DA. FK506 rescues peripheral nerve allografts in acute rejection. J Neurotrauma. (2001) 18:217–29. doi: 10.1089/08977150150502631 11229713

[33] Aral AM Zamora R Barclay D Yin J El-Dehaibi F Erbas VE. The effects of tacrolimus on tissue-specific, protein-level inflammatory networks in vascularized composite allotransplantation. Front Immunol. (2021) 12:591154. doi: 10.3389/fimmu.2021.591154 34017323 PMC8129572

[34] Kato T Tzakis AG Selvaggi G Gaynor JJ David AI Bussotti A. Intestinal and multivisceral transplantation in children. Ann Surg. (2006) 243:756–64. doi: 10.1097/01.sla.0000219696.11261.13 PMC1570576 16772779

[35] Roskott AM Nieuwenhuijs VB Dijkstra G Koudstaal LG Leuvenink HGD Ploeg RJ. Small bowel preservation for intestinal transplantation: a review. Transplant Int. (2011) 24:107–31. doi: 10.1111/tri.2010.24.issue-2 21083772

[36] Muñoz-Abraham AS Patrón-Lozano R Narayan RR Judeeba SS Alkukhun A Alfadda TI. Extracorporeal hypothermic perfusion device for intestinal graft preservation to decrease ischemic injury during transportation. J Gastrointest Surg. (2016) 20:313–21. doi: 10.1007/s11605-015-2986-x 26487331

[37] Moers C Pirenne J Paul A Ploeg RJ. Machine perfusion or cold storage in deceased-donor kidney transplantation. New Engl J Med. (2012) 366:770–1. doi: 10.1056/NEJMc1111038 22356343

[38] op den Dries S Karimian N Sutton ME Westerkamp AC Nijsten MWN Gouw ASH. Ex vivo normothermic machine perfusion and viability testing of discarded human donor livers. Am J Transplant. (2013) 13:1327–35. doi: 10.1111/ajt.12187 23463950

[39] Eshmuminov D Becker D Bautista Borrego L Hefti M Schuler MJ Hagedorn C. An integrated perfusion machine preserves injured human livers for 1 week. Nat Biotechnol. (2020) 38:189–98. doi: 10.1038/s41587-019-0374-x PMC7008032 31932726

[40] Clavien PA Dutkowski P Mueller M Eshmuminov D Bautista Borrego L Weber A. Transplantation of a human liver following 3 days of *ex situ* normothermic preservation. Nat Biotechnol. (2022) 40:1610–6. doi: 10.1038/s41587-022-01354-7 35641829

[41] Hamminger P Rica R Ellmeier W. Histone deacetylases as targets in autoimmune and autoinflammatory diseases. Adv Immunol. (2020) 147:1–59. doi: 10.1016/bs.ai.2020.06.001 32981634

[42] Brilli LL Swanhart LM de Caestecker MP Hukriede NA. HDAC inhibitors in kidney development and disease. Pediatr Nephrol. (2013) 28:1909–21. doi: 10.1007/s00467-012-2320-8 PMC3751322 23052657

[43] Levine MH Wang Z Bhatti TR Wang Y Aufhauser DD McNeal S. Class-specific histone/protein deacetylase inhibition protects against renal ischemia reperfusion injury and fibrosis formation. Am J Transplant. (2015) 15:965–73. doi: 10.1111/ajt.13106 PMC5493154 25708614

[44] Hadden MJ Advani A. Histone deacetylase inhibitors and diabetic kidney disease. Int J Mol Sci. (2018) 19(9):2630. doi: 10.3390/ijms19092630 30189630 PMC6165182

[45] Liu H. The roles of histone deacetylases in kidney development and disease. Clin Exp Nephrol. (2021) 25:215–23. doi: 10.1007/s10157-020-01995-5 PMC7925501 33398599

[46] Golchin A Seyedjafari E Ardeshirylajimi A. Mesenchymal stem cell therapy for COVID-19: present or future. Stem Cell Rev Rep. (2020) 16:427–33. doi: 10.1007/s12015-020-09973-w PMC7152513 32281052

[47] Khoury M Cuenca J Cruz FF Figueroa FE Rocco PRM Weiss DJ. Current status of cell-based therapies for respiratory virus infections: applicability to COVID-19. Eur Respir J. (2020) 55:2000858. doi: 10.1183/13993003.00858-2020 32265310 PMC7144273

[48] Liu S Peng D Qiu H Yang K Fu Z Zou L. Mesenchymal stem cells as a potential therapy for COVID-19. Stem Cell Res Ther. (2020) 11:169. doi: 10.1186/s13287-020-01678-8 32366290 PMC7197031

[49] Zaki MM Lesha E Said K Kiaee K Robinson-Mccarthy L George H. Cell therapy strategies for COVID-19: Current approaches and potential applications. Sci Adv. (2021) 7:eabg5995. doi: 10.1126/sciadv.abg5995 34380619 PMC8357240

[50] Shetty R Murugeswari P Chakrabarty K Jayadev C Matalia H Ghosh A. Stem cell therapy in coronavirus disease 2019: current evidence and future potential. Cytotherapy. (2021) 23:471–82. doi: 10.1016/j.jcyt.2020.11.001 PMC7649634 33257213

[51] Grumet M Sherman J Dorf BS. Efficacy of MSC in patients with severe COVID-19: analysis of the literature and a case study. Stem Cells Trans Med. (2022) 11:1103–12. doi: 10.1093/stcltm/szac067 PMC9672850 36181766

[52] Lu K Geng St Tang S Yang H Xiong W Xu F. Clinical efficacy and mechanism of mesenchymal stromal cells in treatment of COVID-19. Stem Cell Res Ther. (2022) 13:61. doi: 10.1186/s13287-022-02743-0 35130977 PMC8822653

